# Comparison of Postoperative Wound Healing in Fistulectomy and Fistulectomy With Marsupialization in Patients With Low Fistula-in-Ano

**DOI:** 10.7759/cureus.89414

**Published:** 2025-08-05

**Authors:** Ajwa Tariq Aziz, Sidra Shabbir, Hifza Bashir, Aqsa Iqbal, Aneeza Tufail, Marya Yousaf, Muhammad Abdullah, Nizam Fatima, Mirza Zeeshan Sikandar

**Affiliations:** 1 General Surgery, Rashid Latif Medical College, Lahore, PAK; 2 General Surgery, Rahbar Medical and Dental College, Lahore, PAK; 3 Community Medicine, Central Park Medical College, Lahore, PAK; 4 General Surgery, Central Park Medical College, Lahore, PAK; 5 Primary and Secondary Health, Rural Health Center Mandi Ahmedabad, Okara, PAK

**Keywords:** fistula-in-ano, fistulectomy, fistulotomy, marsupialization, randomized controlled trial, wound infection

## Abstract

Introduction: Fistula-in-ano is a relatively common benign disease of the anorectal region, which may pose considerable complications in terms of surgery, as this disease has the propensity to recur and may be accompanied by postoperative wound healing. Surgical therapy can be considered the key in the management, and surgery involves fistulectomy or fistulotomy as a part of surgical treatment. Nevertheless, due to the fear of slow healing of the wound and acquiring infection because of common methods, some other methods have been explored, and some have been suggested to be defined as the process of marsupialization, which could allow decreasing the size of the open wound and stimulate its recovery quicker.

Objective: To compare the incidence of postoperative wound infection between fistulectomy alone and fistulotomy with marsupialization in patients with low anal fistula.

Materials & Methods: The study(randomized controlled trial) was done in Arif Memorial Teaching Hospital and Central Park Medical College, Lahore, from January to September 2024. Low fistula-in-ano patients were randomized into two groups, i.e., Group A (SUR one fistulectomy) and Group B (170 patients in each group). The same surgical team applied all their procedures to reach spinal anesthesia. Wound infection was determined after the operation was done clinically and verified by culture at 10 days of follow-up. Statistical analyses based on SPSS version 26 were carried out, and p < 0.05 was considered significant.

Results: Average age of sample population was 36.3, 12.2 years, with males prevailing (80.9 %). Baseline data were similar between the two groups. All in all, there were 58 patients (17.1%) who had wound infections after the operation. Group B (fistulotomy with marsupialization) resulted in a much lower wound infection rate (10.0%) as compared with group A (24.1%) (p=0.001). The findings presented in subgroup analyses indicated that the benefit of marsupialization was consistent across all age, gender, fistula type, and duration of the disease, with the majority of the differences being statistically significant.

Conclusion: Fistulotomy and marsupialization showed a considerably reduced postoperative wound infection rate compared to the conventional fistulectomy in patients with low fistula-in-ano.

## Introduction

Fistula-in-ano

It is one of the most popular benign diseases of the anus in everyday surgical activity. It can be defined as an epithelialized tract interlinking two surfaces, based on rectal mucus and perianal skin mostly [1]. Fistula-in-ano is an unsound connection generally involving some level of granulation tissue, which runs from the ano-rectal crater (internal opening) to the exterior opening on the skin of the perineum or the buttock. Fistula-in-ano has typically been caused by an anorectal abscess that has ruptured through faulty surgery or spontaneously. Various disease processes, such as tuberculosis, Crohn's disease, malignancy, etc., may be associated with anal fistula [2].

Fistula-in-ano is hard to treat

The treatment of choice is surgery, and the objectives are to drain the infection, destroy the fistulous tract, prevent the disease from recurring or becoming persistent, and maintain the anal sphincters. Other surgical procedures that are available in the treatment of low fistula-in-ano are fistulotomy and fistulectomy [3]. Fistulectomy procedure is the complete removal of the fistulous tract, thus evading the risk of secondary tract evasion and the benefit of entire tissue in the histopathological study [4].

A fistulotomy opens the tract of the fistula, thereby creating smaller unepithelialized wounds. Both fistulotomy and fistulectomy are wounds that leave the raw, unepithelialized endo and perianal tissue that needs to heal over and that can need hospitalization, irrigation, and dressing with a risk of bleeding and recurrent sepsis [5]. Marsupialization of the fistula is the method that reduces the size of the wound, accelerates the process of healing, and enhances continence by minimizing anal deformity without taking excessive time in healing and hospital time [6].

Compared to a fistulectomy, a fistulotomy with marsupialization heals faster and the period of discharge of the wound is shorter, but it does not make the operating time longer [6]. A study showed that wound infection after an operation occurred in 3.3% in fistulotomy plus marsupialization versus 10% in fistulectomy of low fistula in-ano, but the differences were not very significant (p>0.05) [7, 8].

This study is rationale to compare the postoperative wound infection in fistulectomy and fistulotomy, as well as the marsupialization of patients with low fistula-in-ano. Presently, we are operating with fistulectomy as the standard procedure to treat fistula, which leads to long hospitalization and poor wound healing. However, it has been noted through literature that fistulotomy with marsupialization has the capacity to minimize postoperative wound infection and accelerate wound healing as compared to fistulectomy, although findings were non-pronounced. Lately, it has been stated that marsupialization of wounds results in early wound healing. This is the reason we desire to undertake the study because there is no local evidence in this context, since we cannot perform fistulotomy with marsupialization in the local context, rather than doing fistulectomy. However, the outcome of this study would assist us in adopting the method that is more reliable with less risk of infection following the treatment of fistula in-ano.

## Materials and methods

A randomized controlled trial was conducted at the Department of General Surgery at Arif Memorial Teaching Hospital and at Central Park Medical College and Teaching Hospital, Lahore, in association with the Pakistan Health and Research Forum from January 2024 to September 2024. The clinical trial was registered in the National Clinical Trials Registry with the registration number NCT07080424. A sample size of 340 patients (170 patients in each group) was calculated with 5% level of significance and 80% power of the test, taking the expected frequency of postoperative wound infection to be 3.3% with fistulotomy with marsupialization and 10.0% with fistulectomy in patients with low anal fistula [7]. Double blinding was done to ensure the confidentiality of study participants. Patients were selected by Non-Probability, Consecutive Sampling. Patients of either gender aged between 18 and 70 years presenting with fistula in-ano and patients with recurrent fistula, with anal fissures, chronic colitis, and hemorrhoids were excluded.

The patients, 340 of whom met the inclusion criteria, were admitted to the Outpatient Department (OPD) of the Department of Surgery, Arif Memorial Teaching Hospital, Lahore, and Central Park Teaching Hospital, Lahore, after ethical clearance of the hospital’s ethical committee. Each patient who participated in this research gave informed consent. Random sampling was done by buying a lottery ticket to separate the patients into two equal groups. In group A, they performed a surgical procedure called fistulectomy alone. Fistulectomy followed the procedure of marsupialization in group B. Through spinal anesthesia, all the surgeries were conducted by one and the same surgical team, which was assisted by a researcher. Antibiotics of ciprofloxacin and metronidazole were administered at the time of operation. Three additional days after surgery, ciprofloxacin and metronidazole were administered to the patients along with subsequent transfer to post-surgical facilities. In line with the hospital guidelines for patient discharge, patients were released. Follow-up of the patients in the OPD was done after 10 days. Patients were also examined at the end of 10 days with regard to wound healing, but in case of the presence of pus and/ or pain at the wound site, as well as growth of bacteria in the pus, which tested positive on culture test, then the condition was termed to be an infection.

Statistical analysis

The Statistical Package for the Social Sciences (IBM) version 26.0 was used to analyze all the data obtained and put in using SPSS version 26.0. The quantitative variables, such as age and duration of fistula-in-ano, have been reported as a mean +/- S.D. Qualitative variables such as gender, type of fistula, and postoperative wound infection have been provided as frequency and as a percentage. Comparison of postoperative wound infection was also done and compared between the two groups using the chi-square test, and a p-value of 0.05 or less is considered significant. The stratification of data is by age, gender, length of fistula, and type of fistula. A chi-square test has been done by post-stratification, with p-value = 0.05 taken as significant.

## Results

In the study, there were 340 research participants. The participants had a mean age of 36.3 years and a standard deviation of 12.24 years, which implies that the age range of the sample is rather wide. As tabulated in Table 1, most of the participants were aged 18 to 35 years (n=183), with 53.8%, whereas a larger proportion of 34.7% was aged 36 to 53 years (n=118), and 11.5% was aged 54 to 70 years. In regard to gender distribution, the male population formed the major portion of the study population, representing 80.9% (n=275) compared to 19.1% (n=65) representing the female population, which clearly denotes that the disease under study portrays a male preponderance. With respect to fistula type, the participants were almost equal in terms of the intersphincteric type and the low-transsphincteric type of fistula. Precisely, the number of intersphincteric fistulas comprised 50.3% (n=171), and the number of low-transsphincteric fistulas was 49.7% (n=169); thus, of the two subtypes of the fistulas, the two were nearly equal in occurrence within the population under study.

**Table 1:** Baseline Characteristics of Study Population

Characteristics	Participants (n=340)
Age (years)	36.30 ± 12.24
18-35 years	183 (53.8%)
36-53 years	118 (34.7%)
54-70 years	39 (11.5%)
Gender	
Male	275 (80.9%)
Female	65 (19.1%)
Type of Fistula	
Intersphincteric	171 (50.3%)
Low-transsphincteric	169 (49.7%)
Duration of disease (months)	7.60±2.40
3-7 months	183 (53.8%)
8-12 months	157 (46.2%)

It is of moderate chronicity with the average disease duration of 7.6 months\2.4 months. Considering the categorization based on the duration of the disease, the majority of the participants (n=183; 53, 83%) had the 3-7-month result with regard to the duration of disease, with 46.23% (n=157) reporting the duration of 8-12 months resulting in a fairly even distribution of results related to shorter and longer duration of the disease within the inclusion range considered.

The population of the study was randomly separated into two arms, namely: the Fistulotomy with Marsupialization group (n=170) and the Conventional Fistulectomy group (n=170). Expectedly, the review of the baseline characteristics revealed that they were similar between the two groups, and there were no statistically significant differences among them. The age of patients included in the Fistulotomy with Marsupialization group had a mean of 36.36 with a 12.03 standard deviation, whereas the Conventional Fistulectomy group had a mean of 36.24 with a 12.48 standard deviation (p=0.926), and the age was comparable. The number of patients in each group categorically did not show any significant differences between each other (p=0.968) with the highest proportion (53.5% vs. 54.1%) in 18-to-35-year age group, (35.3% vs. 34.1%) in 36 to 53 years age group and (11.2% vs. 11.8%) in 54 to 70 years age group. As far as gender is concerned, the majority of the participants in both groups (Fistulotomy with Marsupialization group and Conventional Fistulectomy group) were detected to be males (81.2 and 80.6), whereas the proportion of females was 18.8% and 19.4%, respectively (p=0.890), which marks a similar profile of gender distribution.

Referring to the type of fistulas, intersphincteric fistulas were found to be somewhat more frequently occurring in both groups (50.6% and 50% in the Fistulotomy with Marsupialization group, respectively and Conventional Fistulectomy group, respectively), but low-transsphincteric fistulas were reported to be equally prevalent at 49.4% and 50% in these two groups, respectively, with no significant difference between groups (p=0.914). The average time taken to become ill was almost equal in both the groups, 7.61 + 2.61 and 7.59 + 2.18 in fistulotomy with marsupialization and Conventional Fistulectomy, respectively (p=0.964). The stratification by disease duration revealed that approximately half of the patients had the disease extent up to 3 to 7 months (54.1% vs. 53.5%) and the rest of the patients up to 8 and 12 months (45.9% vs. 46.5%) in each sample without an important disparity (p=0.913). Generally, the comparison between the two groups proves that the results obtained were comparable at the baseline; that is, in age, gender, type of fistula, and time of year the disease has lasted, which is adequate to justify any form of further comparison regarding treatment outcomes, which is highlighted in Table 2.

**Table 2:** Assessment and Comparison of Baseline Characteristics of Study Groups by Employing the Chi-Square Test

Characteristics	Marsupialization (n=170)	Fistulectomy (n=170)	χ²/t-value	df	P-value
Age (years)	36.36 ± 12.03	36.24 ± 12.48	t = 0.092	338	0.926
18–35 years	91 (53.5%)	92 (54.1%)	χ² = 0.064	2	0.968
36–53 years	60 (35.3%)	58 (34.1%)			
54–70 years	19 (11.2%)	20 (11.8%)			
Gender			χ² = 0.019	1	0.890
Male	138 (81.2%)	137 (80.6%)			
Female	32 (18.8%)	33 (19.4%)			
Type of Fistula			χ² = 0.012	1	0.914
Intersphincteric	86 (50.6%)	85 (50.0%)			
Low-Transsphincteric	84 (49.4%)	85 (50.0%)			
Duration (months)	7.61 ± 2.61	7.59 ± 2.18	t = 0.046	338	0.964
3–7 months	92 (54.1%)	91 (53.5%)	χ² = 0.012	1	0.913

Fifty-eight (17.1%) of the 340 study participants experienced a postoperative wound infection as compared to 282 (82.9%) who did not suffer any wound infection. This suggests that, although the prevalence of overall postoperative wound infection was rather low, a significant percentage of patients were affected by this adverse complication. In Table 3, wound infection rates of both arms of treatment were compared. Since 170 patients underwent fistulotomy with marsupialization, only 17 (10.0%) of them developed a wound infection, compared to 41 (24.1%) patients of the Conventional Fistulectomy group. This deviation was statistically significant (p=0.001), and it is an indication of having a much lower incidence of wound infection in the Fistulotomy with Marsupialization group. This observation implies a positive influence of marsupialization on the wound healing process after the surgery.

**Table 3:** Comparison of Postoperative Wound Infection between the Study Groups by Employing the Chi-Square Test

Wound Infection	Marsupialization (n=170)	Fistulectomy (n=170)	χ²	df	P-value
Yes	17 (10.0%)	41 (24.1%)	11.07	1	0.001**
No	153 (90.0%)	129 (75.9%)			

In Table 4, a close subgroup examination of postoperative wound infection among the several demographic and clinical groupings is given. The age subgroups revealed research tendencies in the fistulotomy with marsupialization: 9.9% of the patients in the age group 18-35 years who had undergone marsupialization had wound infection compared to 23.9% of the conventional group (p=0.011). In the ages 36-53 years, there was an infection rate of 10.0% in marsupialization, as compared to 24.1% in conventional surgery (p=0.041). In the most ancient subgroup (5470 years), the rates of infection with the marsupialization method were lower numerically (10.5% and 25.0%, respectively), and the difference was not found to be statistically significant (p=0.239), which could be associated with smaller numbers. The gender check indicated a highly significant difference in wound infection in the male patients following fistulotomy with marsupialization procedure (10.1%) and conventional fistulectomy (24.1%) (p=0.002). The infection rates among the females were 9.4 vs. 24.2, respectively; however, the difference between the two was not statistically significant (p=0.110).

**Table 4:** Comparison of Postoperative Wound Infection between the Study Groups across Various Subgroups by Employing the Chi-Square Test

Subgroup	Marsupialization n/N (%)	Fistulectomy n/N (%)	χ²	df	P-value
Age					
18–35 years	9/91 (9.9%)	22/92 (23.9%)	6.41	1	0.011*
36–53 years	6/60 (10.0%)	14/58 (24.1%)	4.17	1	0.041*
54–70 years	2/19 (10.5%)	5/20 (25.0%)	1.38	1	0.239
Gender					
Male	14/138 (10.1%)	33/137 (24.1%)	9.61	1	0.002*
Female	3/32 (9.4%)	8/33 (24.2%)	2.56	1	0.110
Type of Fistula					
Intersphincteric	9/86 (10.5%)	21/85 (24.7%)	6.01	1	0.014*
Transsphincteric	8/84 (9.5%)	20/85 (23.5%)	6.01	1	0.014*
Duration of Disease					
3–7 months	9/92 (9.8%)	21/91 (23.1%)	5.95	1	0.015*
8–12 months	8/78 (10.3%)	20/79 (25.3%)	6.00	1	0.014*

Subgroup comparison of fistula type also indicated large advantages of marsupialization. Marsupialized intersphincteric fistula patients had a 10.5% infection rate as compared with 24.7% of patients handled conventionally (p=0.014). In low-transsphincteric fistula, 9.5% and 23.5% patients developed infection (p=0.014). An analysis with subgroups of disease duration showed that in the group of patients with disease duration between 3 and 7 months, the prevalence of infections in the marsupialization group was 9.8% as compared with 23.1% in the conventional group (p=0.015), whereas patients with disease duration of 8 to 12 months showed infection results of 10.3% (marsupialization) versus 25.3% (conventional), respectively (p=0.014).

All in all, these subgroup analyses uniformly showed that the postoperative wound infection rate of patients who underwent the fistulotomy with marsupialization procedure was statistically lower in the vast majority of these stratifications, further providing statistical evidence as to why the former treatment is a much safer procedure as compared to a conventional fistulectomy.

## Discussion

Fistula-in-ano is also a long-standing problem of surgery due to anatomic difficulties, recurrence, and postoperative morbidities, the most common problem being wound infection. The current randomized controlled trial was an assessment of the comparative incidence of postoperative wound infection after fistulotomy and marsupialization compared to conventional fistulectomy as a treatment of low-level anal fistulas. After our study, a much lower wound infection rate postoperatively was seen in those treated with fistulotomy modified by marsupialization (10.0%) as opposed to that of standard fistulectomy (24.1%) (p=0.001). These observations can also be correlated with the fact that marsupialization decreases the extent of the raw wound and increases the rate of epithelization, resulting in the reduced likelihood of bacterial colonization and infection [9,10].

Superiority of marsupialization over any demographic and clinical subgroups, based on age, gender, the kind of fistula, and duration of disease, was also established with the use of subgroup analysis. The situation in the elderly subgroup (54 70 years) was different in that no statistically significant difference was noted, but the trend toward the reduction in the rates of infection was observed in the marsupialization group, indicating a possible beneficial effect even in such a population.

The smaller infection rates possible after marsupialization may be explained by the fact that this operation permits good drainage of the wound, and the dead space is limited, with minimal disturbance of the surrounding tissue. In marsupialization, the edges of the wound are closed with the bottom of the fistulous tract so that the cavity is smaller, well-drained, and this will allow immediate healing of the wounds with the sphincter intact [11, 12]. This procedure will also enable continuous drainage of a wound and reduce the bacterial load, edema, and exudate accumulation, which have been proven to be the factors of surgical site infections [13, 14].

Our findings are comparable to those of other studies in the region and other parts of the world. They found that wound infection occurrence after fistulotomy with marsupialization was 8.3% against 23.1% after fistulectomy ( p=0.013), which suggests that marsupialization helps to avoid wound sepsis [15]. Equally, Jain et al. established evidence of quicker healing of wounds and low rates of postoperative infectiousness of marsupialization involving the use of saturation level when compared with the conventional operation of fistulectomy [16]. This has been confirmed in meta-analysis studies of lower infection rates, speedier return to work, and similar recurrence with marsupialization [17,18].

However, one should recognize that there exist limitations. The follow-up in our work was quite short, and we did not evaluate the long-term recurrence or comorbidities, including diabetes, hypertension, smoking, hygiene, and incontinence rates, as it is actually one of the determinants of the overall efficiency of fistula surgery.

Formation of bias

There were also some limitations in that despite the bias that occurred because of the paucity of selection that was caused by randomization, since these were ones done in a two-center setting, this may pose a challenge to generalizability, and subsequent multicenter studies would therefore be significant in reaffirming these findings across the varied backgrounds.

## Conclusions

This randomized controlled trial proved that the use of fistulotomy with marsupialization procedure supplanted the normal fistulectomy procedure in patients with low anal fistula, as there are remarkably fewer chances of post-surgical wound infection with regard to fistulotomy with marsupialization. The results always preferred the marsupialization in the majority of the demographic and clinical subgroups, which indicates its possible benefits in the mitigation of wound complications and higher healing rates. Marsupialization seems to produce a more favorable environment and a lower bacterial load, hence resulting in fewer infections and perhaps a quicker recovery by shrinking the size of the raw wound, enhancing drainage, and limiting tissue trauma.
